# Distance and size matters: A comparison of six wildlife camera traps and their usefulness for wild birds

**DOI:** 10.1002/ece3.4240

**Published:** 2018-06-25

**Authors:** Christoph Randler, Nadine Kalb

**Affiliations:** ^1^ Department of Biology Eberhard Karls University Tübingen Tübingen Germany

**Keywords:** bird research, camera or photo traps, methods check, passive infrared sensor, wildlife trail cameras

## Abstract

Camera traps are increasingly used in ecological research. However, tests of their performance are scarce. It is already known from previous work that camera traps frequently fail to capture visits by animals. This can lead to a misinterpretation of ecological results such as density estimates or predation events. While previous work is mainly based on mammals, for birds, no data about if and how camera traps can be successfully used to estimate species diversity or density are available. Hence, the goal of our study was an empirical validation of six different camera traps in the field. We observed a total number of *N* = 4567 events (independent visits of a bird) in 100 different sessions from March 2017 until January 2018 while camera traps were deployed. In addition, *N* = 641 events are based on a comparison of the two close‐up camera traps especially designed for birds. These events were all directly observed by the authors. Thus, the cameras can be compared against the human observer. To give an overall assessment and a more generalizable result, we combined the data from the six camera traps and showed that bird size category (effect size = 0.207) and distance (effect size = 0.132) are the most important predictors for a successful trigger. Also, temperature had a small effect, and flock size had an impact with larger flocks being captured more often. The approach of the bird, whether it approached the camera frontally or laterally had no influence. In Table 8, we give some recommendations, based on our results, at which distances camera traps should be placed to get a 25%, 50%, and 75% capture rate for a given bird size.

## 1 INTRODUCTION

Camera traps are increasingly used in ecological and conservation research to, for example, estimate animal density and to assess population size (Fancourt, 2016; McCallum, 2013; Rovero, Zimmermann, Berzi, & Meek, 2013; Silveira, Jácomo, & Diniz‐Filho, 2003). Camera traps can be a helpful tool in animal research as its appropriate usage allows a short‐ as well as long‐term observation of animals and their behavior without the presence of an actual observer. Thus, such camera traps can observe a given plot 24 hr on 7 days, which cannot be covered by observers. Also, these camera traps are time‐saving because they release images when an animal passes by. This is achieved by a passive infrared trigger, that is, the camera detects moving objects (in usual cases animals) that have a temperature difference to the ambient environment. Therefore, it is less time‐consuming than watching hours of instantaneous video recordings because less material is sampled. However, traps also can be triggered even when an animal does not pass by (e.g., vegetation movement, changes in temperature) while cameras also may fail to trigger an animal.

Furthermore, camera traps might reduce observer bias as the event of interest is captured on a photo that can be evaluated by multiple persons after the experiment. Hence, camera trapping in place and instead of traditional wildlife survey methods has become common despite inherent flaws in equipment and a dearth of research to test their fit for purpose (Meek & Pittet, 2012). In the same line of argument, Després‐Einspenner et al., (2017) noted that empirical validations of conventional monitoring methods remain nearly nonexistent, although these studies would uniquely allow researchers to evaluate the accuracy of density estimates and the practical applicability of commonly used survey methods. This was one of the main reasons that informed our current work. Some studies tried to test the quality of different camera traps, either in comparison with other methods or in a comparison among different camera types or models.

### 1.1 Comparison of different camera models

For example, Swann, Hass, Dalton, and Wolf (2004)—based on model animals to emulate wildlife—compared six different camera models from different brands. Driessen, Jarman, Troy, and Callander (2017) stated that animal detections vary among commonly used camera trap models and all camera models failed to detect a substantial proportion of the total known triggers and visits by animals. Dixon et al. (2009) compared video and passive vs. active camera systems and reported a higher sensitivity of the passive infrared systems. Urlus, McCutcheon, Gilmore, and McMahon (2014) also showed that different camera models had different rates of positive triggers. Swan, Di Stefano, and Christie (2014) by comparing two different camera models showed that the results would be significantly different depending on the camera model used. Weingarth, Zimmermann, Knauer, and Herich (2013) tested six digital camera models with regard to trigger speed and image quality, with a special focus to identify lynx (*Lynx lynx*). Thus, there are already some camera model comparisons available, but the models and brands are steadily technically improving, and also, no study yet compared cameras with a focus on birds.

### 1.2 Comparison of different survey techniques

To assess the quality of camera traps, some studies compared different types of recordings vs. automatic triggered systems. Lyra‐Jorge, Ciocheti, Pivello, and Meirelles (2008) compared track plots with cameras and found that camera traps enabled a better determination of species while the track plots were better in registering individuals. Glen and Dickman (2003) reported that camera trapping was less open to misinterpretation than sand plots (especially in adverse weather conditions). A comparison between live trapping and camera traps was carried out by De Bondi, White, Stevens, and Cooke (2010). These authors showed that camera trapping is similar to live traps and they suggest using camera traps for small mammals because it is less costly. Similarly, Driessen and Jarman (2014) suggested using camera traps compared to live trapping because it is a more time‐efficient method of detecting differences and monitor changes in abundance. In this present study, we compare the camera traps with an observer being present during the time when the cameras were deployed. This is an important aspect because comparing one method against another gives only the relative merits of one approach over the other and cannot indicate which of these methods has the best relationship with reality (Ballard et al. 2014). However, one approach that is most similar to ours was done by Jumeau, Petrod, and Handrich (2017). These authors compared permanent recordings (video) with triggered pictures at two meters distance. The camera traps failed to capture 43.6% of small mammals, such as voles and mice, and 17% of medium‐sized mammals (such as hare, fox, or badger).

### 1.3 Variables influencing positive triggers

Some confounding variables that affect the efficiency of camera traps have been identified in different studies. One is the ambient temperature or the difference between the temperature of the animal and the environment. Camera traps with a passive infrared sensor measure “heat in motion,” that is they compare the ambient temperature with the temperature of the moving object. Most studies reported that the camera traps worked better at lower temperatures (e.g., Swann et al., 2004). As mammals and birds are endotherm animals, their body temperature remains more or less fixed. If the ambient temperature is low, for example, below 10°C, the difference to the animals is large and the cameras have a higher probability to detect the animal. If the ambient temperature is more or less similar to the temperature of the animals, the cameras trigger the animal with lower probability (Welbourne, 2014 reports a required difference of 4–5°C).

In addition, body mass is considered an important predictor with larger or heavier animals being recorded more frequently (e.g., Anile & Devillard, 2016; Lyra‐Jorge et al., 2008; Tobler, Carrillo‐Percastegui, Leite Pitman, Mares, & Powell, 2008; Urlus et al., 2014). This is based on the fact that the heat signature increases with increasing body size.

Similarly to body size, distance is an important predictor for the same reasons (see above). Heat signature becomes smaller with an increasing distance, and thus, farther distant animals are detected with a lower probability.

Frontal vs. lateral approach has been an additional variable that has—to our knowledge—never been addressed in the literature. However, given the specifications of the cameras and the fact that a frontal approaching animal covers a smaller area than a lateral approaching one, it could be that camera traps are better at detecting a movement that goes parallel to the tested cameras, rather than a movement approaching from a frontal direction. Some previous tests on humans showed that this might be the case (unpublished experiments). Therefore, we considered this variable as important for this study.

### 1.4 Studies on birds

However, most studies focused on mammals and only 11.9% on birds (Burton et al., 2015; O'Brien & Kinnaird, 2008). In addition, most studies on birds focused on larger species, such as fowl (Griffiths & Lewis, 2014). Thus, we wanted to assess, if camera traps can be used for bird research, and especially under which conditions. Birds are usually much smaller than most mammals and especially lighter at the same size, better insulated by their feathers and mostly quicker in their behavior and movements.

## 2 AIMS

The main aim was to compare different cameras for bird research with an observer present and to assess the influence of bird size and distance on detection/trigger probability. We conducted observations with the camera traps outdoors and watched the animals simultaneously to collect field data (always with a human observer being present). We selected camera types that were especially designed for the observation of birds (see below), as well as other cameras that—supposed from their technical data—might be suitable for bird studies. In addition, we pooled the data across all six camera traps (using the mean of successful triggers) to get a generalizable result to give advice where to set up a camera trap to capture bird species of different sizes. We hypothesize that bird size and distance have a significant influence on the positive triggers, with larger and nearer birds being detected at a higher probability.

## 3 METHODS

### 3.1 Camera trap types

We selected six different cameras based on the data of the brand (mainly trigger speed, detection distances, and sensitivity) and information on websites (see, e.g., https://www.trailcampro.com; https://www.wildkamera-test.com/) or recommended by other scientists. The camera systems chosen fulfilled the following requirements: apparently good picture quality according to the manufacturer data (similar to Weingarth et al., 2013), trigger speed about ≤1.5 s, <€ 300 costs. Detection distances should be ≥10 m, and a burst function to make up to three images without delay. The selection is based on four typical camera traps and two cameras especially designed for capturing birds at lower distances (Table 1). All cameras used a passive infrared sensor. We tested one camera of each model because we assumed that the quality within a sold charge of cameras is homogenous (see Weingarth et al., 2013).

**Table 1:** Infrared camera traps used in the study (approximate price in early spring 2017)

Camera traps (suited for all purpose)	Approx. price in €	Trigger speed (s)	Max. detection distance (m)	View angle	Sensor
Spypoint Force 11D	241	0.07	24–30	35°	PIR
SecaCam Raptor	148	0.4	20	52°	PIR, 5 MP
Bushnell Trophy Cam Aggressor 48 No Glow (model 119777)	284	0.2	33.5	n.a.	PIR
Dörr SnapShot Extra Black 5.0 black (model 204401)	260	1.2	15	52°	PIR, 12 MP
Camera traps especially designed for birds					
Bushnell Natureview (model 119740)	282	0.2	30.5	n.a.	PIR
Wingscapes BirdCam Pro (model WCB 00119)	267	≈ 1.5	12.2	40°	PIR

We set up different feeders with different kinds of food such as sunflower seeds, peanuts, apples, and mealworms for a variety of birds on trees, rocks and stones, and on the ground. Furthermore, we provided water in some places to attract birds for drinking and bathing. In addition, we used natural observations of animals without food, for example, at lakes when ducks and swans were passing by. We studied birds from the size of a winter wren (*Troglodytes troglodytes*) which is the second smallest bird species in Europe (about 10 cm, 8–10 g), up to the size of captive greater rheas (*Rhea americana;* 120–140 cm; 20–25 kg).

**Figure 1:**
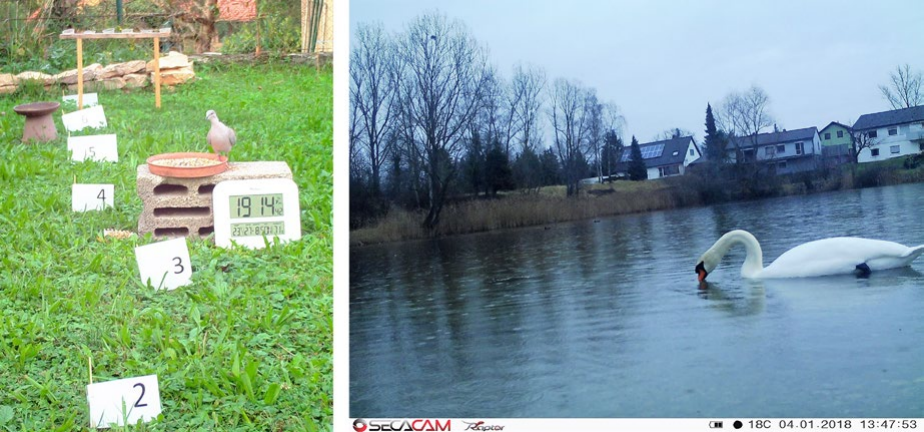
Examples of two settings, (a) baited at the feeder with food at different distances. (b) Mute Swan (*Cygnus olor*) swimming laterally to the camera in a natural unmanipulated situation

The feeders were set up prior to the camera tests. After a habituation period of some days, we build‐up the cameras, retracted for at least 20 m, and started the observation immediately. During the whole observation period, every bird that visited the feeder was noted (labeled event, following Meek et al., 2014), down to the species level as well as clock time (sometimes supported by an extra watch). If food was distributed across a wider area, we used signposts to measure the distances (see Figure 1). Distances were measured with a rule meter or with a Nikon Aculon AL11 distance meter. The Nikon Aculon AL11 measures distances between 5 and 500 m with a precision of ± 1 m. The Aculon was used when the birds were passing by in an unmanipulated non‐feeding situation (e.g., Figure 1). In cases where no food or water was provided, we observed birds that were passing by from one side to the other, and that were within a predefined angle of about 25° within the core center of the detection area. During the whole time of the camera setup, a human observer was always present and observed the birds. Thus, all events have been verified by an observer, and we compare the camera traps against the real data. We did not compare any events among the cameras without being present. We studied birds at 29 different sites. A session was defined as every situation where the cameras were placed at a specific place and a specific distance. When the distance was changed or the area was changed, a new session was defined (i.e., every time when the cameras where moved a new session started). In total, we made 117 sessions throughout the year, and per session, we observed on average 39 events (range: 1–222). Total observation time was 175 hr.

**Figure 2:**
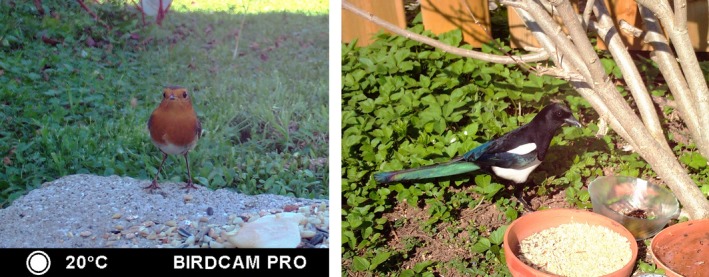
Different modes of approach: Frontal approach (left; Robin *Erithacus rubecula*) vs. lateral approach (right; Magpie *Pica pica*)

In addition, flock size was noted if animals occurred in groups (otherwise the number 1 was assigned). Also, we assessed if animals approached in a lateral or frontal direction, but this was only assigned, when it was clearly visible. Most birds approached the feeder in different angles or turned quickly after arriving so that the movement could not be assigned to a frontal or lateral approach. Figure 2 shows the frontal vs. a lateral approach. The temperature given in the respective cameras was also noted for every event when a picture was taken. A total sample of *N* = 4,567 events was collected, from March 2017 until January 2018 to get the full range of temperatures in Central Europe. These events were used for the general results on distance categories and size classes. Out of these, *N* = 3,641 events could be used for the camera comparison because in these 3,641 cases all cameras have been working simultaneously. All studies were carried out around the vicinity of Tübingen, SW Germany, Central Europe (48°31′17.891″N 9°3′27.521″E). Tübingen has a typical central European climate with about 9°C annual average temperature. The mean temperature measured with the camera traps was 14.23 ± 10.54 (*SD*) and ranged from −1°C to 47°C.

### 3.2 Comparison of cameras designed for close‐up photography

In the close‐up test, we sampled additional *N* = 641 datasets based on the two close‐up cameras (*N* = 317 up to 0.46 m; *N* = 325 up to 0.60 m). BirdCam and Natureview are especially designed for close‐up photography. The special study was done between 22.9.2017 and 4.11.2017. We chose the distances of 46 cm and 60 cm because for these distances, special lenses have been available for the Natureview. For the BirdCam, it was possible to adjust the focus to these distances. We followed the same protocol as described above. All comparisons were made with food supply.

### 3.3 Setup of the cameras

**Figure 3:**
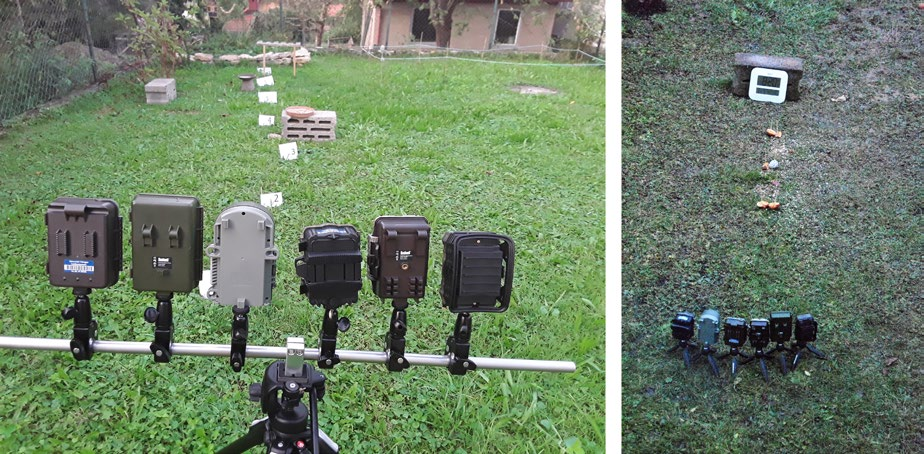
Example of the camera setup on a higher tripod (Manfrotto 055, with head 128 RC) (left), and on small, separate tripods Manfrotto MTPIXI‐B [S/N RA499304] (right)

All cameras were setup simultaneously to capture the same event and make them comparable. Camera traps were either setup on small tripods (Manfrotto MTPIXI‐B [S/N RA499304]) when the position was low. Alternatively, the cameras were mounted onto a larger tripod (Manfrotto 055, with a head 128 RC) when the cameras had to be placed on a higher position (see Figure 3). The position, where a camera was placed, was assigned randomly, all cameras were placed in a way that the feeder was in the middle of the picture. We used the same type of batteries (Varta Alkaline AA, 4006 LR6, Mignon, MN1500) and 16 GB SanDisc card in each camera. Burst function was enabled in about 50% of the data (in all cameras simultaneously; *N* = 2558) and disabled in another 50% (simultaneously in all cameras; *N* = 2009). The recovery time was set to zero. The camera traps were all set on the highest infrared sensitivity. Then, the cameras were setup and the observation started. The session ended when the camera position or its distance to the feeder was changed. We systematically varied the distances at which the cameras were placed. When cameras were placed at a feeder, the different distance categories were covered, that is, the first session started between 1 and 1.5 m, the next session covered another distance category etc.; another day, the first session started at 5 m and then categories were changed to reflect all distance categories in a sufficient manner (with sufficient events). Also, we intentionally used larger distances at which the cameras made no photos to reliably assess the detection distances.

In some instances, one of the cameras made no photos, for example, because the battery was empty or there were problems with the *SD* card. These events were treated as missing data for the respective camera and not used for the camera comparisons, but for the overall results. This led to *N* = 3,641 total events where all cameras worked simultaneously without failure.

### 3.4 Statistical methods

All statistical analyses were performed with SPSS 24.0. An event was defined as every visit of a bird at a feeder. Some species, such as tits visit feeders only for a short period of time (e.g., often below 5 s) but have some visits during the same minute, so two visits can be easily separated and were treated as different events. Every event was coded. 0 was assigned when a bird was observed by the observer, but the camera did not trigger the bird and 1 was assigned when the camera triggered the bird. This was defined as successful trigger. The observer was able to identify all species in the field. We did not survey the quality of the images, and the decision was always based on whether the bird species was identifiable, and not whether the images were of a good quality because the main aim in behavioral research is the identification of different species. However, on some images, the bird that was captured was not identifiable (although the species identity was noted by observation). Then, the value 0 was assigned (no successful trigger). If species were visible only partly, but species identification was possible from the image, 1 was assigned. For the comparison among all cameras, a Cochrane test was used. For subsequent comparison between two cameras, we used sign tests.

We grouped the bird species into eight different size categories: up to the size of a blue tit (*Cyanistes caeruleus*), about finch size (e.g., great tit *Parus major,* chaffinch *Fringilla coelebes,* house sparrow *Passer domesticus*), blackbird size (e.g., blackbird *Turdus merula*), magpie size (crow, *Streptopelia decaocto,* jay *Garrulus glandarius*), mallard size (*Anas platyrhynchos*), and larger sized birds (goose *Anser anser*, Mute Swan *Cygnus olor*, greater rhea *Rhea americana;* see Table 2). We set up and measured distances in a variety of distances, but afterwards grouped the distances into different categories: up to 1, 1.5, 2, 2.5, 3, 4, 5, 7, 10, and 50 m (Table 2). These different size classes were needed because birds were rather small, and we wanted to assess at what distance a given bird size class could be successfully triggered with a given probability. Thus, amalgamating size and distance categories might lose a lot of information.

**Table 2:** Size classes and distance categories with sample sizes

	Sample size
Size class	
Up to blue tit size	881
Finch size	2,514
Blackbird size	368
Magpie size	605
Duck size	100
Goose size and larger	99
Distance categories	
Up to 1.00 m	567
Between 1.01 and 1.50 m	670
Between 1.51 and 2.00 m	707
Between 2.01 and 2.50 m	760
Between 2.51 and 3.00 m	546
Between 3.01 and 4.00 m	621
Between 4.01 and 5.00 m	479
Between 5.01 and 7.00 m	86
Between 7.01 and 10.00 m	107
Between 10.01 and 50.00 m	24

We combined the data across all camera traps to get a more generalizable result and to analyze the impact of body size, distance, and temperature. The calculation of models for every camera type or model separately provides only information about the specific camera model. Our goal, however, was to provide information that might also be applicable to other camera types that have not been used during our study. Hence, we decided to calculate the percentage of cameras with a positive trigger of an event to receive results that may be generalizable to other products or models. Therefore, we calculated the positive triggers of an event based on all cameras and concerted it to a percentage, which ranged from 0 to 100 (if none or all cameras took a picture). Thus, it is a percentage of the number of cameras with a positive trigger on an event. These percentages were arcsine square‐root transformed to make the data suitable for a general linear model. The flock size was ln‐transformed after the addition of 1 (ln of [flock size + 1]).

Then, we used a general linear model with size class and distance as fixed factors, temperature and flock size as covariates. For the statistical calculations, we used the transformed data, but for the figures, these were back‐transformed to percentages.

## 4 RESULTS

### 4.1 Comparisons of all six camera traps

In general, the number of successful triggers was between 17.2% and 31.7%. The number of successful triggers differed significantly between camera models (Cochrane's Q = 560, *df* = 5, *p* < 0.001). Sign tests revealed significant difference between SecaCam and BirdCam and between BirdCam and Spypoint. There was no difference between Spypoint and Trophy, but between Trophy and Dörr as well as between Dörr and Natureview (Table 3).

**Table 3:** Comparison and ranking of the number of successful bird triggers between camera models. Total number of events *N* = 3,641. Sign test was used to test differences between pairs of cameras in order of ranking

Camera type	Rank	Number successful triggers	Percentage (%)	Comparison with next type	*p*
				Test statistic	
SecaCam	1	1,154	31.7	−2.415	0.016
BirdCam	2	1,084	29.8	−8.429	<0.001
Spypoint	3	844	23.2	−0.295	0.768 ns
Trophy	3	835	22.9	−3.848	<0.001
Dörr	4	724	19.9	−3.840	<0.001
Natureview	5	627	17.2		

Note

Ns indicates not significant.

In addition, we have split the total file into different distance categories to assess whether the cameras perform differently in different distances (Table 4; based on a Friedman test). Here, the SecaCam performed best at the lowest distances but the performance decreased with increasing distance. BirdCam performed best at the distances between 2 and 5 m. Spypoint showed a good performance at 1 m and at 50 m. Trophy showed best results at larger distances (4 m, 5 m). Dörr showed the best results at 3 m, while Natureview generally scored low. There were no significant differences at the 7 and 10 m category. This indicates that there are fewer differences at larger distances.

**Table 4:** Ranking of camera models across distance categories based on number of successful bird triggers. Within each distance category, Friedman test was used to determine significant differences in the number of successful bird triggers between camera models

Camera Type	1 m	1.5 m	2 m	2.5 m	3 m	4 m	5 m	7 m^a^	10 m^a^	50 m	Median
SecaCam	1	1	2	2	2	2	3	–	–	3	2
BirdCam	3	3	1	1	1	1	1	–	–	3	1
Spypoint	2	4	5	3	4	4	5	–	–	1	4
Trophy	5	2	3	5	5	3	2	–	–	2	3
Dörr	4	6	4	4	3	6	6	–	–	3	4
Natureview	6	5	6	6	6	5	4	–	–	3	5.5
Chi‐square	246.79	117.12	131.60	168.13	61.88	82.06	74.03	1.27	5.00	12.22	
*p*	<0.001	<0.001	<0.001	<0.001	<0.001	<0.001	<0.001	0.938	0.416	0.032	

Note

a Data for 7 and 10 m are not provided because they were not significant.

Similarly, we spilt up the size categories to detect differences (Table 5; based on a Friedman test). SecaCam detected smaller birds best. BirdCam was second on small birds but performed worse on larger sized birds. Spypoint and Trophy were best at detecting larger birds. Dörr had its best performance in the largest birds.

**Table 5:** Ranking of camera models across size categories based on number of successful bird triggers. Within each size category, Friedman test was used to determine significant differences in the number of successful bird triggers between camera models

Camera Type	Up to blue tit	Finch size	Blackbird size	Magpie size	Duck size	Goose/swan	Median
SecaCam	1	1	1	2	1	3	1
BirdCam	2	2	2	1	5	5	2
Spypoint	3	4	3	3	3	1	3
Trophy	4	3	4	4	2	1	3.5
Dörr	5	5	4	5	4	2	4.5
Natureview	5	6	5	6	6	4	5.5
Chi‐square	93.034	288.649	26.223	219.810	31.297	10.771	
*p*	<0.001	<0.001	<0.001	<0.001	<0.001	0.056	

### 4.2 Close‐up test

**Figure 4:**
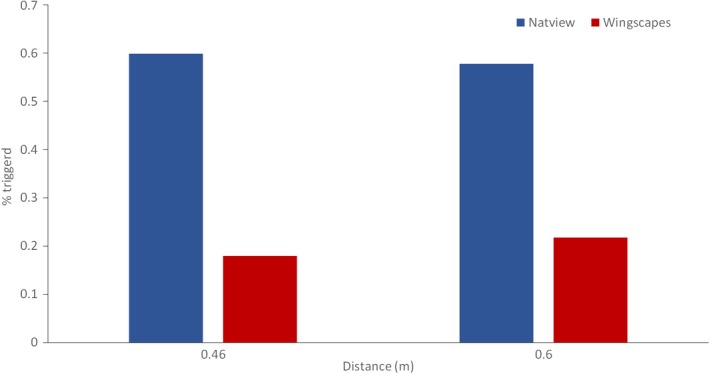
Comparison of number of successful triggers by birds between two close‐up cameras set to take images at two distances (BirdCam and Natureview)

Natureview had more successful triggers compared to BirdCam (Sign test: *Z* = −14.188, *p* < 0.001), both at the 0.46 m distance (*Z* = −9.398, *p* < 0.001) and the 0.60 m distance category (*Z* = −9.317, *p* < 0.001; see Figure 4).

### 4.3 Overall mean results

There was a significant influence of size class, distance, temperature, and flock size on the number of successful triggers (Table 6). The larger the size class and the closer the distance, the higher was the percentage of successful triggers. Larger flock sizes resulted in more successful triggers. Higher temperature was related to higher successful triggers.

**Table 6:** General linear model of the percentage of triggers by birds (mean overall results across all cameras) showing the partitioning of variation and tests of size class and distance and their interactions. Flock size and mean temperature are covariates. Dependent variable is the mean percentage of successful triggers (arcsine square‐root transformed). (*N* = 4,567)

Source	*df*	Mean of squares	*F*	Sig.	Partial eta‐squared
Corrected Model	59	42.267	110.116	<0.001	0.590
Constant	1	96.586	251.632	<0.001	0.053
Flock size	1	11.579	30.166	<0.001	0.007
Temperature	1	13.764	35.858	<0.001	0.008
Size class	5	90.346	235.376	<0.001	0.207
Distance category	9	29.223	76.133	<0.001	0.132
Size class*Distance category	43	3.716	9.682	<0.001	0.085
Error	4,507	0.384			
Total	4,567				
Corrected total variation	4,566				

**Figure 5:**
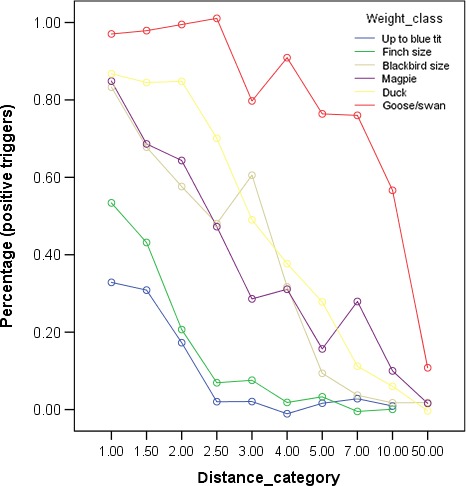
Successful triggers in percent according to weight class and distance

The overall explained variance was high (corrected *R* ^2^ = 0.585). The size class*distance interaction was significant (Figure 5) which shows that the number of successful triggers depends on a combination of a species’ size and its relative distance to the camera. While larger species usually can be detected farther away than smaller species, the interaction shows that at large distance the cameras detect all birds poorly, whereas at close distances large birds are better detected than small birds.

In a subsample of the events, we were able to classify whether a bird approached frontally or laterally (Table 7). We observed 593 frontal and 431 lateral approaches.

**Table 7:** General linear model of the number of triggers by birds showing the partitioning of variation and tests of approach position (frontal vs. lateral), size class and distance and their interactions. Flock size and mean temperature are covariates. Dependent variable is the mean percentage of releases (arcsine square‐root transformed)

Source	*df*	Mean of Squares	*F*	Sig.	Partial eta‐squared
Corrected model	58	8.082	30.660	<0.001	0.648
Constant	1	1.574	5.971	0.015	0.006
Flock size	1	1.348	5.114	0.024	0.005
Temperature	1	0.605	2.294	0.130	0.002
Size class	5	10.996	41.712	<0.001	0.178
Distance category	9	13.490	51.175	<0.001	0.323
Position	1	.002	.007	0.933	0.000
Size class*Distance category	20	1.520	5.767	<0.001	0.107
Size class*Position	2	0.073	.277	0.758	0.001
Distance category*Position	6	0.504	1.911	0.076	0.012
Size class*distance category*Position	11	0.230	0.872	0.568	0.010
Error	965	0.264			
Total	1,024				
Corrected total variation	1,023				

**Figure 6:**
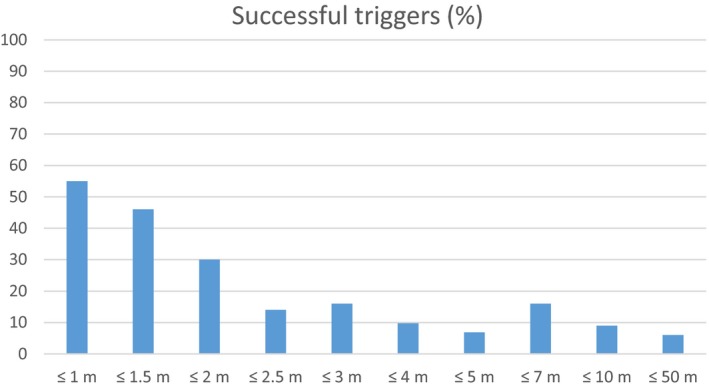
Successful triggers according to distance categories (across all bird sizes)

Again, size and distance were the main predictors. However, frontal vs. lateral approach was not significant. Temperature received no significance, which can be owed to the sample size or the fact that the temperature ranges were smaller in the frontal–lateral approach data subset (*SD* = 7.29) compared to the full data set (*SD* = 11.09). For a more species‐specific analysis, we show in the Appendix also the probability for successful triggers (Appendix). Finally, Figure 6 shows the average percentage of successful triggers across all cameras and all bird size categories. These vary from about 60% at 1 m down to around 5% at 50 m.

## 5 DISCUSSION

This study systematically tested the functionality of camera traps for bird research. While wildlife trail cameras may be suitable for bird research, we put a special emphasize on the distances and size classes as a camera can only deliver reliable results if its detection range is suitable for the focal species of a study. In line with our hypotheses, we found that size and distance are related to successful triggers. Also, larger flocks were triggered with a higher probability compared to single individuals. Temperature had an opposing effect, while frontal vs. lateral approach showed no significance. These aspects are all related to the size or area of the animal because a larger heat signature should be triggered with a higher probability. A larger bird, as well as a nearer bird covers a larger area. Similar, a flock produces a larger heat signature compared to singles.

Distance was a strong and significant predictor of successful triggers (see Figure 6). This is an expected result because the area covered by an animal increases with increasing distance. However, most studies did not vary distances but made the comparisons usually on one or a few distance categories. In this respect, our study is novel.

Bird size was another important predictor of successful triggers. This is in line with previous findings on other species, especially in mammals (Anile & Devillard, 2016; Lyra‐Jorge et al., 2008; Tobler et al., 2008; Urlus et al., 2014).

Temperature had a significant influence on positive triggers. However, positive triggers were higher in higher ambient temperatures, which first seems counter‐intuitive, but birds in a warmer environment may be more mobile and agile compared to birds in colder environments when they try to save energy by moving less.

Frontal vs. lateral approach was not significant. This contrasts with expectations, but these expectations are based on mammals and the body size and shape vary greatly between mammal species and birds. In addition, the body shape of individuals depends on its behavior in front of the camera, that is, the body position and shape of a marten that feeds on the ground differs from a marten climbing onto tree or standing on its hind limbs. In contrast to mammals that usually have a more cuboid form, birds sometimes appear in a more spherical shape. Also, when birds approach, they spread their wings before landing so that there might be no big difference between frontal and lateral approach in the area covered within the sensor field. As the sample size for the frontal and lateral approaches is only *N* = 1024, this might have an influence on the detectability. However, if it does not reach significance with such a sample size, we suppose that approach position has no practical relevance, at least in birds.

The interaction between size class and distance shows that it does not follow a simple pattern. However, it shows that at short distances, there are well‐defined differences between the size classes while at larger distances, the differences between the size classes are diminished. Bird behavior might also have an influence, for example, different species may use their wings during an approach in a different way, which may influence the detection probability. Furthermore, the birds may approach in different manners, for example, by regular small hops or by larger jumps or with different approach speeds, or they may differ in their time they remain in the detection area. This might be the case in great tits that usually approach quick and leave quick if they collect a sunflower seed, in comparison to great tits that sit at a bird fat ball for sometimes a minute.

The average number of successful triggers was about 20% (ranging from 17.2% to 31.7%). Although this could be considered low, one must keep in mind that we systematically varied the distances up to such large distances where no photos could have been taken. This was done to reliably estimate the maximum detection range. Compared to Glen, Cockburn, Nichols, Ekanayake, and Warburton (2013), the successful triggers were low. These authors—working on small‐ to medium‐sized mammals—reported a rate of 80%–90% successful triggers in slowly moving stoats (*Mustela erminea*) and of 70–80% in hedgehogs (*Erinaceus* spec.).

This study has several strengths. First, we observed and tested the cameras nearly for 1 year, reflecting all different climate aspects in central Europe. Second, we sampled birds in all categories from small winter wren up to a flightless rhea, which also adds to the generalizability of our findings.

*Miscellaneous*: The time stamp of some cameras did not always include seconds; therefore, we suggest for all camera manufacturers to print the seconds in the time format to make differences more easily to assess.

## 6 CONCLUSION AND IMPLICATIONS

Generally, camera traps can be used for bird research, but with a strong emphasize on keeping distances low (Figure 5). As main conclusion, we suggest that researchers should use Table 8 to place the camera at a specific distance. In Table 8, we give raw estimate at which distance a species should be depicted with an average camera.

**Table 8:** Estimates of successful trigger probability in a given size class based on the field data (see Figure 5)

	25% chance of capture (m)	50% chance of capture (m)	75% chance of capture (m)
Up to blue tit size	1.7	–	–
Finch size	2	1.2	–
Blackbird size	4.4	2.5	1.3
Magpie size	4.5	2.5	1.3
Duck size	5.1	3	2.4
Goose size and larger	≈25	≈10	7

## CONFLICT OF INTEREST

Both authors have no conflict of interest to declare. The cameras were bought by ourselves, and not supplied by the company.

## DECLARATION

All authors on the paper have seen and approved the submitted version of the manuscript and have substantially contributed to the work. All persons entitled to co‐authorship have been included. CR and NK designed the study, CR made most of the observations, CR and NK analyzed and discussed the data, both contributed to the writing of the manuscript.

## DATA ACCESSIBILITY

The data are archived in Dryad (DOI: https://doi.org/10.5061/dryad.693b3f4).
